# Supplementary material for: MetaRibo-Seq measures translation in microbiomes
Source: Nat Commun. 2020 Jun 29;11:3268. doi: 10.1038/s41467-020-17081-z (PMC7324362; doi:10.1038/s41467-020-17081-z)
Supplement: Supplementary file 10 — Supplementary Data 7 [file 41467_2020_17081_MOESM10_ESM.zip › File2/Confidence_High_Taxonomy/292148_out.krona.html]

Javascript must be enabled to view this page.

members
magnitude
magnitudeUnassigned
count
unassigned
taxon
rank

292148\_out

5

2759
5
superkingdom

kingdom
4751
5

451864
5
subkingdom


SRS023914\_contig\_number\_6354SRS051031\_contig\_number\_contig-100\_417.199373SRS098571\_contig\_number\_45342
5204
phylum
5
3

452284
2
subphylum

1538075
2
class

order
2
162474

742845
2
family

55193
2
genus

species
2
55194

SRS013687\_contig\_number\_contig-100\_1338.141312SRS1041145\_contig\_number\_contig-100\_7739.137510
